# RNA as a feasible marker of *Trypanosoma cruzi* viability during the parasite interaction with the triatomine vector *Rhodnius prolixus* (Hemiptera, Triatominae)

**DOI:** 10.1371/journal.pntd.0010535

**Published:** 2022-07-07

**Authors:** Paula Finamore-Araujo, Gabriel Lucio Silva da Fonseca, Cecília Stahl Vieira, Daniele Pereira de Castro, Otacilio Cruz Moreira

**Affiliations:** 1 Real Time PCR Platform RPT09A, Laboratory of Molecular Virology, Oswaldo Cruz Institute, Oswaldo Cruz Foundation, Rio de Janeiro, Brazil; 2 Laboratory of Biochemistry and Physiology of Insects, Oswaldo Cruz Institute, Oswaldo Cruz Foundation, Rio de Janeiro, Brazil; 3 Postgraduate Program in Science and Biotechnology, Biology Institute, Federal Fluminense University, Niterói, Brazil; Bose Institute, INDIA

## Abstract

A recurring question concerning *Trypanosoma cruzi* DNA detection/quantification is related to the fact that DNA amplification, by itself, does not differentiate between viable or dead parasites. On the other hand, RNA can be considered a potential molecular marker of pathogens viability. Herein, we developed a quantitative real-time PCR with reverse Transcription (RT-qPCR) to quantify viable *T*. *cruzi* in artificially infected *Rhodnius prolixus* whilst evaluating differences between DNA and mRNA quantification along the insect midgut during 5, 9, 15 and 29 days after feeding. The RT-qPCR presented an improved performance with linearities ranging from 10^7^ to 10^2^ parasites equivalents and 3 to 0.0032 intestine unit equivalents, and efficiencies of 100.3% and 102.8% for both *T*. *cruzi* and triatomine targets, respectively. Comparing both RT-qPCR and qPCR, we confirmed that RNA is faster degraded, no longer being detected at day 1 after parasite lysis, while DNA detection was stable, with no decrease in parasite load over the days, even after parasite lysis. We also observed statistical differences between the quantification of the parasite load by DNA and by RNA on day 15 after feeding of experimentally infected *R*. *prolixus*. When assessing different portions of the digestive tract, by RT-qPCR, we could detect a statistically significant reduction in the parasite amount in the anterior midgut. Oppositely, there was a statistically significant increase of the parasite load in the hindgut. In conclusion, for this study parasite’s viability in R. prolixus digestive tract were assessed targeting *T*. *cruzi* mRNA. In addition, differences between DNA and RNA detection observed herein, raise the possibility that RNA is a potential molecular viability marker, which could contribute to understanding the dynamics of the parasite infection in invertebrate hosts.

## Author summary

In this study, we developed and standardized a Real-Time PCR with Reverse Transcription (RT-qPCR) to determine *T*. *cruzi* viability in *R*. *prolixus* samples. Moreover, we aimed to assess differences between the amplification signals of DNA and mRNA on a *T*. *cruzi* colonization kinetics in experimentally infected *R*. *prolixus*. Thus, it was possible to analyze the potential of parasite’s RNA as a molecular viability marker in parasite-vector interaction. This novel RT-qPCR methodology has potential application in viability assessment and raises the possibility for further monitoring of the parasite load in infected insects or studies related to vectorial capacity. Furthermore, the analysis of parasite viability by RT qPCR could be an especially effective tool for Chagas disease diagnostic purposes.

## Introduction

Chagas disease is a neglected tropical illness, caused by the flagellated protozoan *Trypanosoma cruzi*, which involves a major social and economic problem in several countries in Latin America. It is estimated that six to seven million individuals are infected, mainly in Central and South America [1]. Moreover, although it is considered a neglected disease, Chagas disease has a worldwide prevalence and its presence in several countries out of the endemic ones has turned it into a global health problem in recent years [2]. This parasitosis caused by *T*. *cruzi* infection evolved from a primitive enzooty and became a human health problem, due to many factors such as population migrations and increased human changes in the environment [3].

*T*. *cruzi* is represented by a group of isolates, which comprises clones and strains, presenting great heterogeneity in biological behavior [4]. Currently, seven genotypes, or Discrete Typing Units (DTUs) are recognized (TcI-TcVI and TcBat) and identifiable by specific molecular markers [5]. In addition, *T*. *cruzi* has a complex biological cycle, as it develops between vertebrate hosts, including several domestic and sylvatic mammal species, and strictly hematophagous insect vectors belonging to the subfamily Triatominae (Hemiptera: Reduviidae) [6]. During its biological cycle, *T*. *cruzi* alternates between four main development stages which can be found in both vertebrate and invertebrate hosts, such as, bloodstream and metacyclic trypomastigotes, infective but nonreplicative stages, as well as amastigotes and epimastigotes, noninfective but replicative stages. In addition to these four main forms, *T*. *cruzi* have many intermediate developmental stages. These different stages are morphologically differentiated by the position of the kinetoplast in relation to the nucleus and the region of emergence of the flagellum. And, besides morphological differences, several changes in the expression of some stage-specific genes can also occur during metacylogenesis [7,8].

Regarding the diagnosis of *T*. *cruzi* in vertebrate and invertebrate hosts, molecular methodologies have been proposed as an alternative for an accurate detection of *T*. *cruzi* infection, as well as making possible the examination of degraded or small volumes samples [9]. DNA-based PCR methods for *T*. *cruzi* detection in different biological samples have shown greater sensitivity, specificity, and reproducibility than the examination through optical microscopy, considered the classic method of analysis [10,11]. Therefore, PCR or qPCR assays are useful for qualitative or quantitative diagnosis for Chagas disease and genotyping parasite diversity to monitor the distribution of *T*. *cruzi* DTUs in different endemic regions [9].

Currently, there has been interest in distinguishing DNA and RNA amplification signals of several pathogens for viability assessment [12–15], specially due to the established short half-life of RNA molecules after cell death compared to DNA molecules [16,17]. However, a recurring question regarding the quantification of *T*. *cruzi* through its DNA detection extends to the fact that it is not clearly elucidated if the presence of DNA is related to the detection of viable infectious parasites. In this context, developing a highly sensitive molecular assay can offer significant advantages for identification and quantification of *T*. *cruzi* viability. Furthermore, analyzing parasite viability may contribute to better investigations of the parasite infection dynamics in invertebrate hosts and vector competence. It can also contribute to quality surveillance of commercialized food, since testing food samples for viable *T*. *cruzi* can add to a better understanding and control of oral transmission outbreaks.

In the present study, we developed and standardized a Real-Time PCR with Reverse Transcription (RT-qPCR) to determine *T*. *cruzi* viability in *R*. *prolixus* samples. Moreover, we aimed to assess differences between the amplification signals of DNA and mRNA on a *T*. *cruzi* colonization kinetics in experimentally infected *R*. *prolixus*. Thus, it was possible to analyze the potential of parasite’s RNA as a molecular viability marker in parasite-vector interaction.

## Methods

### Trypanosoma cruzi culture

Strains and clones of *Trypanosoma* cruzi from DTUs I to VI subpopulations, represented by Dm28c (TcI), Y (TcII), INPA 3663 (TcIII), INPA 4167 (TcIV), LL014 (TcV) and CL (TcVI), were obtained from Coleção de Protozoários da Fundação Oswaldo Cruz, Rio de Janeiro, Brazil (Fiocruz, COLPROT, http://www.colprot.fiocruz.br). Epimastigotes forms were maintained and grown in brain heart infusion (BHI) medium (Sigma-Aldrich) containing hemin and supplemented with 10% heat-inactivated foetal bovine serum at 28°C for 4 days to reach late-log growth phase. Parasites were harvested by centrifugation (3000× g for 10 min at room temperature), washed three times with 0.15 M NaCl, 0.01 M phosphate buffer pH 7.2 (PBS), prior to insect infection and DNA extraction. The number of parasites was determined by counting in a Neubauer hemocytometer using an optical microscope and expressed as parasites/mL.

The isolation of Dm28c (TcI) trypomastigote and amastigote forms was carried out using VERO cells [18,19]. Briefly, cell cultures were infected with mice-derived bloodstream trypomastigotes, in a 10:1 parasite/host cell ratio. Meanwhile, infected cells were maintained at 37°C in a 5% CO_2_ atmosphere. After 5–6 days, the supernatant was collected, centrifuged at 500 ×g for 10 min, and held for 30 min at 37°C for the migration of trypomastigotes into the supernatant. The amastigotes were recovered in the pellet.

### Preparation of *T*. *cruzi* lysates

1 x 10^6^ *T*. *cruzi* epimastigotes/mL were harvested by centrifugation as previously described, and lysates were prepared by immersing the samples in boiling water for 20 minutes. After cooling, parasite lysates were maintained at 28°C and 300 μL aliquots were taken at different time points post-lysis (0, 1, 2 and 3 days after lysis) for further use in DNA extraction and RNA extraction. The presence of living parasites was examined using a Neubauer chamber, under light microscopy at a 200X magnification.

### *Rhodnius prolixus* maintenance, feeding and infection

*R*. *prolixus* were reared and maintained in an insectary at Laboratório de Bioquímica e Fisiologia de Insetos, Instituto Oswaldo Cruz (IOC/Fiocruz), Rio de Janeiro, under controlled temperature (28°C) and relative humidity (60–70%) conditions. For the experiments, fifth instar nymphs starved for 30 days were chosen and then fed with defibrinated and heat-inactivated rabbit blood through a latex membrane of an artificial apparatus [20]. Only fully engorged insects were selected for the assays.

*T*. *cruzi* infection was evaluated during three independent feedings using rabbit blood containing epimastigote forms of *T*. *cruzi* Dm28c clone (1 x 10^7^ parasites/mL). These three biological replicates were performed 30 days apart between them. Parasite quantification (detailed below) was analyzed in two experimental replicates through three different methods, reverse transcription quantitative PCR (RT-qPCR), quantitative PCR (qPCR) and counting on Neubauer chamber under an optical microscope.

For the dissection procedure, we used scissors to cut a small part of the final portion of the Connexivum allowing the dissection of the digestive tract. With tweezers, the digestive tract was gently pulled from the hindgut and the separation of the different compartments of the digestive tract was then performed in a Petri dish. To identify the anatomical landmarks we followed the figure of the digestive tract presented in the review paper published by Kollien & Schaub [21].

The entire digestive tract of *R*. *prolixus* nymphs was dissected individually on days 5, 9, 15 and 29 after feeding. Immediately after each dissection, five entire digestive tracts were gathered in a pool (n = 5) and stored together with 180 μL of TE Buffer (Tris-EDTA 1X Solution, pH 8.0) for extraction. Moreover, the anterior midgut (AM), posterior midgut (PM) and hindgut (H) of infected *R*. *prolixus* were also dissected separately on days 5, 9, 15 and 29 after feeding, and gathered in a pool (n = 5). Each pool consisted of the corresponding portion of the digestive tract (AM, PM or H) dissected from five individuals and grouped together into a 1.5 mL tube with 180 μL of TE Buffer (Tris-EDTA 1X Solution, pH 8.0) for extraction.

To validate our protocol and exclude the possibility of contamination, a control experiment was performed with non-infected insect group fed on uninfected blood.

### Quantitative multiplex real-time PCR (qPCR) assays

Each pool, containing the corresponding of 5 digestive tracts of dissected *R*. *prolixus*, were pretreated for 2 h at 56°C with 100 μl lysis buffer containing 10 mM Tris-HCl (pH 9.2), 1 mM EDTA and 150 μg/ml proteinase K (Sigma-Aldrich, St. Louis, MO, USA). DNA was purified from the lysate using QIAamp DNA mini kit (Qiagen, Hilden, Germany) and resuspended from the silica column in a final volume of 100 μL of elution buffer from the kit [11]. DNA was stored at– 20°C until further analysis.

Multiplex qPCR reactions were carried out in a final volume of 20 μl, containing 2 μl DNA (8–10 ng), 2× FastStart TaqMan Probe Master Mix (Roche applied science, Mannheim, Germany), 600 nM cruzi1/cruzi2 primers and 250 nM Cruzi3 probe (FAM/NFQ-MGB) targeting *T*. *cruzi* nuclear satellite DNA (SAT-DNA), 300 nM P2B primer, 500 nM P6R primer and 150 nM Triat Probe (VIC/NFQ-MGB) (Applied Biosystems) targeting the 12S ribosomal subunit gene of triatomines [11,19,22]. Sequences of both sets of primers and probes are presented in **Table 1**. The cycling conditions were as follows: 50°C for 2 min, 95°C for 10 min, followed by 40 cycles at 95°C for 15 s and 58°C for 1 min. Amplifications were performed in the ABI Prism 7500 Fast (Applied Biosystems).

**Table 1:** Primer sets and probe sequences for qPCR and RT-qPCR assays.

Targets	Primers/Probes	Sequences	Amplicon size	References
*T*. *cruzi* satellite DNA (Sat-DNA)	Cruzi 1 (Forward)	ASTCGGCTGATCGTTTTCGA	165 bp	[22]
	Cruzi 2 (Reverse)	AATTCCTCCAAGCAGCGGATA		
	Cruzi 3 (Probe)	FAM-CACACACTGGACACCAA-NFQ-MGB		
Triatomine 12S rRNA	P2b (Forward)	AAAGAATTTGGCGGTAATTTAGTCT	163 bp	[11]
	P6R (Reverse)	GCTGCACCTTGACCTGACATT		
	Triat (Probe)	VIC-TCAGAGGAATCTGCCCTGTA-NFQ-MGB		
*T*. *cruzi* GAPDH	TcGAPDH Fw (Forward)	GTGCGGCTGCTGTCAACAT	100 bp	[19]
	TcGAPDH Rv (Reverse)	AAAGACATGCCCGTCAGCTT		

### Parasites standard calibration curves and parasite load normalization

Standard calibration curves for *T*. *cruzi* and triatomine targets were constructed by serially diluting total DNA obtained from non-infected triatomine intestine samples spiked with 10^6^ *T*. *cruzi* epimastigotes (Dm28c clone, TcI). The resulting DNA was serially diluted to a range of 10^6^ to 0.5 *T*. *cruzi* equivalents and 5 to 0.002 triatomine intestine unit equivalents.

Samples parasite load (total parasite equivalents/intestine unit equivalents) was performed by normalizing absolute quantification. That is, dividing the amount of parasite equivalents by the amount of intestine equivalents.

### *In silico* analysis of TcGAPDH primers specificity

The oligonucleotides designed to the *T*. *cruzi* GAPDH gene (TcGAPDH, Silva-Gomes et al., 2014) [19] that code for the protein glyceraldehyde-3-phosphate dehydrogenase (Table 1) were evaluated *in silico* for their specificity. A primer-BLAST search using the sequences of the TcGAPDH oligonucleotides in the “Refseq mRNA” set of the NCBI database was performed with no organism name or group indicated. The primer specificity stringency allowed was at least 2 total mismatches to unintended targets, including at least 2 mismatches within the last 5 base pairs at the 3’ end.

### Quantitative reverse transcription PCR (RT-qPCR) assays

Each pool, containing the corresponding of 5 digestive tracts of dissected *R*. *prolixus*, were eluted in 1mL of TRIzol Reagent (Life Technologies, USA) and incubated for 5 min at 15 to 30°C for the dissociation of the nucleoprotein complex. After homogenizing the samples with TRIzol Reagent, 200 μl of chloroform was added followed by an incubation for 2 to 3 min at 15 to 30°C. After incubation, samples were centrifuged at 2 to 8°C at a speed of 10000g for 18 minutes to separate the upper aqueous phase (containing samples RNA) from interphase and organic phase. The aqueous phase was transferred to a new tube containing an equal volume of 100% ethanol, for RNA precipitation. After that, RNA was purified following RNAeasy Mini Kit (QIAGEN, USA) manufacturer’s instruction.

In other words, total RNA from samples were extracted using TRIzol (Life Technologies, USA) and RNAeasy Mini Kit (QIAGEN, USA), a system that combines methods of purification using a monophasic solution of phenol and guanidine isothiocyanate (TRIzol) and silica-membrane RNeasy spin columns (RNeasy Mini Kit, QIAGEN, USA). RNA quantity and purity were estimated by spectrophotometry at 260/280/230 nm.

Prior to reverse transcription, the purified RNA was pretreated with Amplification Grade Dnase I kit (Sigma-Aldrich, USA) to remove any contaminant DNA. Synthesis of cDNA was carried out with a Superscript III First-strand System (Invitrogen, USA) using 5μL of RNA (not normalized by the mass) following the manufacturer’s protocol for cDNA synthesis with random primer. No RT controls (-RT controls) were included in all cDNA synthesis.

RT-qPCR Real-time assays were performed in ABI Prism 7500 fast sequence detection system and reactions were carried out in a final volume of 20 μL, containing 2 μL cDNA, Power SYBR Green PCR Master Mix (Applied Biosystems, USA) 300 nM TcGAPDH Fw and 300 nM TcGAPDH Rv, targeting T. cruzi GAPDH constitutive gene (Silva-Gomes et al., 2014) [19]; 300 nM P2B and 300 nM P6R (Moreira et al., 2017) [11], which is the same primer set targeting the 12S ribosomal subunit gene of triatomines used in the present paper to detect *R*. *prolixus* DNA. **Table 1** presents sequences of both sets of primers and probes. The conditions for the RT-qPCR were as follows: 95°C for 10 minutes, followed by 45 cycles at 95°C for 15 seconds and 62°C for 1 minute. To monitor the specificity of the primers, a melting curves analysis was carried out after each experiment, resulting in a single peak for each target.

### Parasites standard calibration curves and parasite load normalization

Standard calibration curves for viable parasites were constructed by serially diluting cDNA obtained from non-infected *R*. *prolixus* intestine samples spiked with 10^6^ *T*. *cruzi* epimastigotes. The curve consists of a serially dilution ranging from 10^6^ to 0.5 viable *T*. *cruzi* equivalents and 5 to 0.002 triatomine intestine unit equivalents.

Samples parasite load (viable parasite equivalents/intestine unit equivalents) was performed by normalizing absolute quantification. That is, dividing the amount of parasite equivalents by the amount of intestine equivalents.

### Statistical analyses

All experiments were performed at least in biological triplicates and experimental duplicates and data are reported as quantity mean ± standard deviation. All statistical tests were conducted using the Sigmaplot Windows program version 13.0 (Systat Software, Inc., California, USA). Student’s t test or Mann-Whitney Rank-Sum test was adopted to analyze the statistical significance of the apparent differences, according to the parametric or non-parametric distribution of the data. A p-value less than 0.05 was considered statistically significant (p<0.05). Three correlation analysis between RT-qPCR and microscopy, qPCR and microscopy, and qPCR and RT-qPCR techniques were evaluated using Pearson Product-Moment Correlation (or Pearson Correlation Coefficient), with a range of values from ± 1.0.

## Results

### Standardization of RT-qPCR for absolute quantification of viable *T*. *cruzi*

**Fig 1:**
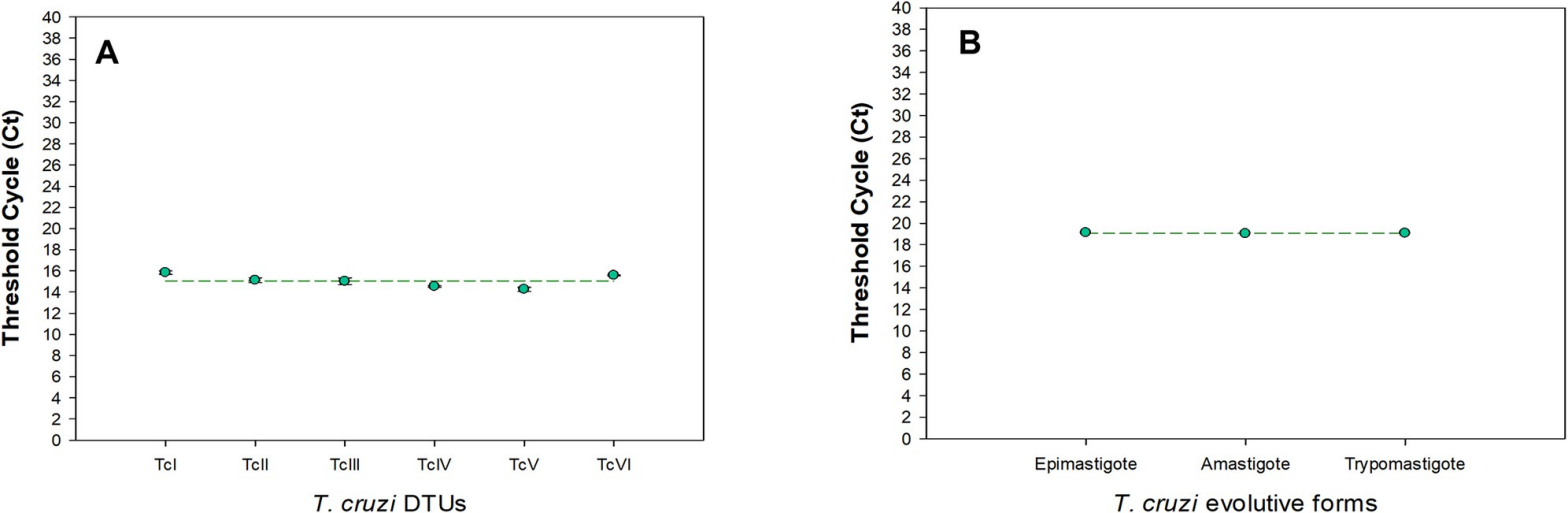
Evaluation of TcGAPDH as a housekeeping gene for RT-qPCR assays. As endogenous housekeeping reference gene, *T*. *cruzi* GAPDH gene was evaluated in different *T*. *cruzi* strains, belonging to DTU I to VI (A) and in different evolutive forms (B). PCR assays were in triplicate, and data were pooled.

Firstly, we developed and standardized a molecular methodology able to detect and quantify *T*. *cruzi* mRNA in *R*. *prolixus* samples. Considering samples of uninfected *R*. *prolixus*, no amplification was observed for the primers targeting *T*. *cruzi* satellite DNA. In addition, the same was observed for the Triatomine 12S rRNA primers, targeting the *R*. *prolixus*, which only amplified the samples containing the insect’s RNA. Furthermore, both sets of primers showed no dimer formation after the amplification phase, in the melting curve test (**S1 Fig**). For the absolute quantification of viable parasites, through RT-qPCR, *T*. *cruzi* GAPDH (TcGAPDH) was selected as a target, since it is a housekeeping gene expressed constitutively in all parasite evolutive stages and in all the six DTUs (**Fig 1**), which can be observed by the similarity of Ct values among the samples. In the samples from epimastigotes of the 6 DTUs, the mean Ct value was 15.06, with 0.60 of standard deviation. In the samples from different evolutive forms (Dm28c amastigotes, epimastigotes and trypomastigotes) the mean Ct value was 19.08, with 0.16 of standard deviation. No significant statistical difference was observed in any comparison. In addition, the TcGAPDH primers specificity to the *T*. *cruzi* GAPDH target amplification was analyzed *in silico*, as described in the Methods section. The Primer-Blast search, using the sequences of the TcGAPDH oligonucleotides in the “Refseq mRNA” set of the NCBI database (with no organism limitation), revealed that the TcGAPDH primers amplifies only for the *Trypanosoma cruzi* glyceraldehyde 3-phosphate dehydrogenase (XM_808702.1, XM_807045.1, XM_029379878.1), always generating a 100 bp PCR product. No unintended PCR products, of any size, were identified in this analysis.

**Fig 2:**
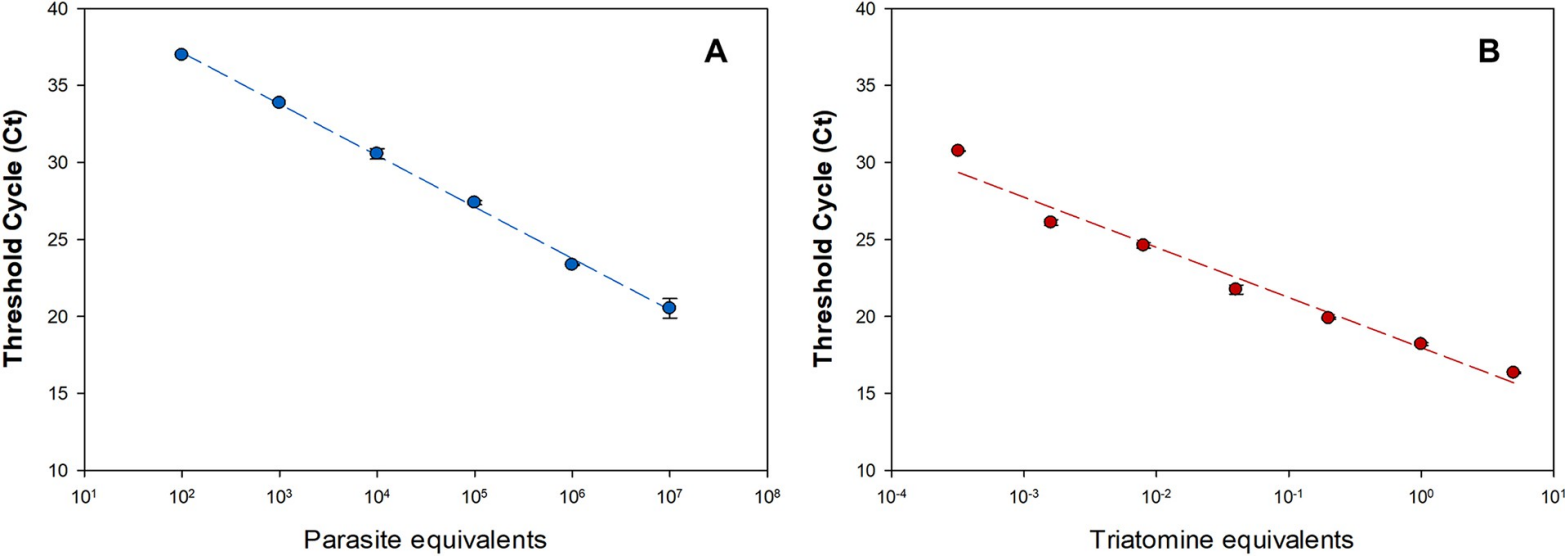
Reportable Range for detection and quantification of viable *T*. *cruzi* in *Rhodnius prolixus* digestive tube by real-time RTqPCR. The Table 2 indicates the standard curve parameters of the assays performed with distinct set of primers. Ten-fold serial dilutions of cDNA were used to generate the standard curve for each target. (A) Linearity ranging from 10^6^ to 10 parasite equivalents, for the TcGAPDH target (B) Linearity ranging from 3 to 0.0032 triatomine equivalents, targeting the gene correspondent to the 12S region of the ribosomal RNA of triatomines.

Following our methodology, detection of *T*. *cruzi* cDNA was linear from 10^7^ to 10^2^ parasite equivalents (**Fig 2A**). Under these conditions, it was possible to obtain a high coefficient of determination (R^2^ = 0.99) for TcGAPDH RT-qPCR. Moreover, *R*. *prolixus* cDNA detection was linear, ranging from 3 to 0.0032 intestine unit equivalents (**Fig 2B**) and the R^2^ was 0.99 for triatomine 12S rRNA gene RT-qPCR (**Table 2**). The results confirmed the improved performance of the methodology for both targets, which were able to detect and quantify parasite mRNA in *R*. *Prolixus* samples.

**Table 2:** Standard curve parameters with distinct set of primers.

Primers	Linear Coefficients^[1]^		r^2^^[2]^	Amplifcation
	Slope	Intercept		Efficiency (%)
A.TcGAPDH	-3,32	39,71	0,99	100,3
B.Triatomine 12S rRNA	-3,26	13,23	0,98	102,8

[1] Linear coefficients from y = ax + b, where a is the slope and b is the intercept

[2] Standard curves coeficient of determination.

### Comparison between quantification of total parasites and viable parasites, by qPCR or RTqPCR

**Fig 3:**
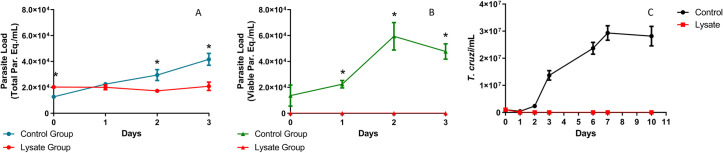
Comparison between DNA and RNA detection of viable and non-viable *T*. *cruzi* samples. A) Quantification of *T*. *cruzi* DNA by qPCR; B) Quantification of *T*. *cruzi* RNA by RT-qPCR; C) *T*. *cruzi* quantification by microscopical examination in a growth curve, with parasite counting using a NEUBAUER Chamber. The symbols corresponding to the control (viable) and lysate (non-viable) groups are indicated in each graph. Data represent analysis from an experiment with n = 3. Data were analyzed using student t-test. *p<0,05.

In order to investigate if RNA is a potential molecular marker to assess *T*. *cruzi* viability, we compared the ability of the TcGAPDH RT-qPCR assay to detect viable *T*. *cruzi* cells with a TaqMan qPCR multiplex assay previously developed to quantify genomic DNA of triatomines infected with *T*. *cruzi* (Moreira et al., 2017) [11]. In **Fig 3**, the time during which both molecules can be detected in samples containing live parasites (control group) and in samples containing heat-treated lysed *T*. *cruzi* (lysate group) was investigated. With this comparison between viable and non-viable parasites, we assessed whether it was possible to perform differentiated quantification by both qPCR and RT-qPCR.

When parasite load was evaluated using qPCR (**Fig 3A**) we observed that DNA detection remains stable for the lysate group over a period of days (0–3 days of incubation), while the control group demonstrated a statistically significant increase in DNA detection over the same period of days. At the same time, parasite load assessed by RT-qPCR (**Fig 3B**) demonstrated no TcGAPDH mRNA detection for the lysate group over days of incubation, as the control group showed an increase in TcGAPDH mRNA during the same period of days. In comparison, we used light microscopy to assess *T*. *cruzi* growth since live and lysed parasites were placed in a culture medium over the days after lysis to evaluate parasites survival and viability of these two groups (**Fig 3C**). The results of RT-qPCR agreed with microscopic examination, in which both were negative, in all days of incubation, for the lysate group, while the results of qPCR were positive.

### Monitoring of experimental *T*. *cruzi* infection in *R*. *prolixus* after feeding

**Fig 4:**
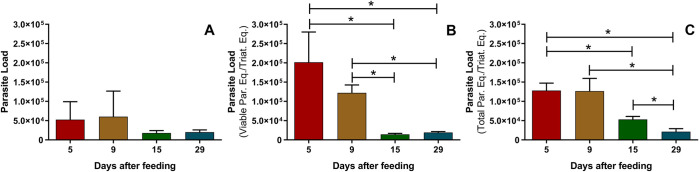
Comparison between three methods for parasite load quantification from insect samples under increasing periods after feeding. A) Microscopical examination, with parasite counting using a neubauer Chamber; B) Quantification of *T*. *cruzi* RNA by RT-qPCR; C) Quantification of *T*. *cruzi* DNA by qPCR. Analysis (n = 3) from a pool of 5 digestive tubes of *R*. *prolixus* under increasing periods after feeding. Data were analyzed using student t-test. *p<0,05.

As the parameters for quantifying the parasite load were defined by DNA and RNA detection, we assessed the parasite load of *R*. *prolixus* experimentally infected with *T*. *cruzi* (**Fig 4**). The entire digestive tract of the insects was dissected, and parasite load was analyzed, by qPCR and RT-qPCR, and normalized by the amount of *R*.*prolixus* DNA or cDNA present in each sample. In addition, quantification using these molecular methodologies were correlated with parasite counting under optical microscopy. When we performed Pearson’s correlation coefficient analysis, it was not possible to establish a statistical association between RT-qPCR and microscopy, qPCR and microscopy, and qPCR and RT-qPCR methodologies (P> 0.050). In other words, differences in the results obtained between molecular quantification and microscopic examination were ascertained, suggesting the sensitivity of both qPCR and RT-qPCR concerning the parasite quantification over optical microscopy. As it is possible to observe in **Fig 4A**, the parasite loads, estimated by counting with Neubauer chamber, were lower when compared to the other two methodologies. On the other hand, when comparing the quantification results obtained by qPCR and RT-qPCR, we observed that both showed a decrease in the parasite load on day 9 after feeding. However, the lack of correlation between qPCR and RT-qPCR was probably due to differences observed in relation to DNA and RNA quantification, mainly 15 days after feeding. *T*. *cruzi* RNA detection (**Fig 4B**) showed a more pronounced decay in the parasite amount on day 9 to day 15 after feeding. Meanwhile, *T*. *cruzi* DNA (**Fig 4C**) did not change significantly from day 9 to day 15 after feeding. In addition, the quantification of mRNA on days 15 and 29 are lower than the values observed, on the same days, by DNA quantification.

### *T*. *cruzi* infection analysis in different portions of *R*. *prolixus* digestive tract

**Fig 5:**
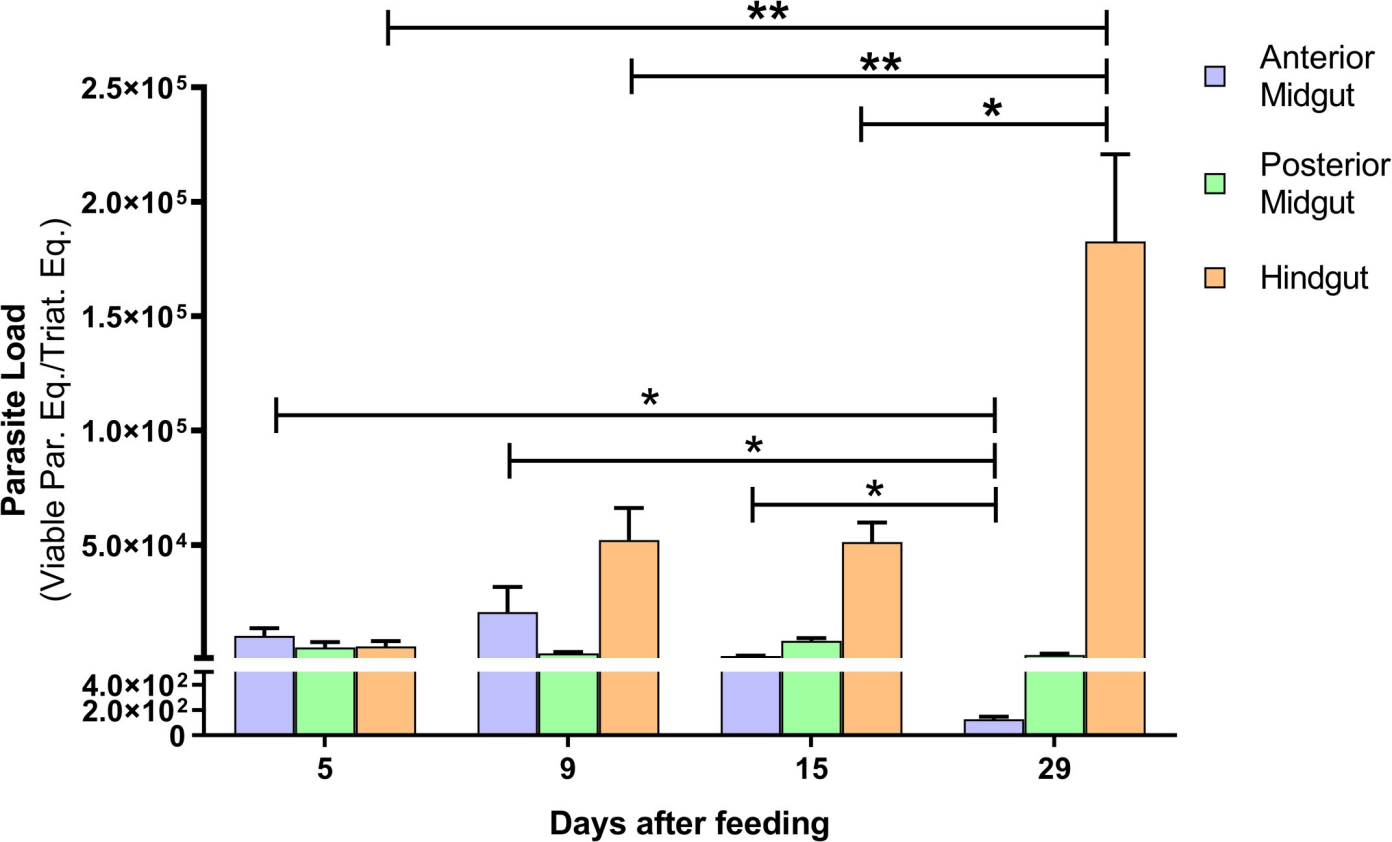
Quantification of viable parasite load by RT-qPCR in different portions of the digestive tract from *R*. *prolixus* experimentally infected. Follow-up of parasite development in a pool of 5 anterior midguts (AM), 5 posterior midguts (PM) and 5 hindguts (H) of *R*. *prolixus* under increasing periods after feeding. Each time point represents three independent experiments (n = 3). Data were analyzed using student t-test. *p<0,05 and **p<0,01.

In order to characterize the infection dynamics, we also assess the parasite load in different segments of *R*. *prolixus* digestive tracts. *T*. *cruzi* quantification was assayed through mRNA detection and normalized according to the amount of *R*. *prolixus* RNA present in each sample (**Fig 5**). The mRNA quantification results revealed that *T*. *cruzi* endures in the PM without showing statistically significant differences between the days. In AM segment, parasite load reduction only becomes statistically significant on day 29 after feeding. On the other hand, in the hindgut portion, a statistically significant increase of the parasite load is observed gradually from day 5 to day 29 after feeding. It is noteworthy that, concomitantly to the increase in the number of parasites from the hindgut over the days, *T*. *cruzi* population from the AM decreased significantly from day 5 to day 29 after feeding.

## Discussion

The classical method to evaluate *T*. *cruzi* natural infection in triatomines is the microscopical examination of dissected intestinal contents. However, it is a laborious and time-consuming procedure that may generate inconclusive and discordant results [10]. Thereby, molecular diagnostic tests have been developed in the last years for rapid detection and quantification of *T*. *cruzi* DNA in different biological samples [9]. The novelty for this study is that we sought to evaluate RNA as a potential marker of *T*. *cruzi* viability in experimentally infected *R*. *prolixus* samples through a methodology based on a SYBR-green RT-qPCR.

Products present in the digestive tract of triatomines, derived from the digestion of blood, can be co-purified with DNA. This can lead to an inhibition of the qPCR reaction, affecting its efficiency. Therefore, it is very likely that *T*. *cruzi* infection rates of triatomines could be underestimated if analyzed by qPCR [23]. The use of an internal amplification control is important to avoid false-negative results, which can occur with highly complex material [24–26], and to correct and normalize DNA variations between samples that are subject to differences in size between species [11]. Previous studies have used housekeeping genes of R. *prolixus* [27] and exogenous heterologous sequences [28] as internal normalization of triatomine samples. To estimate the parasite load in *R*. *prolixus* we utilized a precise multiplex TaqMan qPCR technique, targeting the 12S subunit ribosomal RNA gene as an internal control [11]. The triatomine 12S rRNA target was chosen for the present study since it was previously demonstrated by Moreira et al. (2017) [11] that DNA from different triatomine species presented amplification signal with no significant difference between the adults of all the species analyzed. And even though the triatomine 12s rRNA target was tested only with *R*. *prolixus* for RT-qPCR standardization herein, it is important to validate the RNA quantification technique testing the 12S subunit ribosomal RNA gene with other triatomine species and nymphs from all developmental stages in the future.

Although DNA amplification methods have been largely reported [29], PCR by itself does not differentiate between live and dead parasites due to the high stability of DNA and its persistence following parasite death [15,16,30,31]. Presently, our results showed that *T*. *cruzi* DNA detection remained stable over 3 days after cell lysis, while parasite load assessed by RT-qPCR demonstrated no amplification for the lysate group. Supporting our results, it has been previously observed that DNA of *Leishmania* parasites can remain detectable in scars of cutaneous leishmaniasis patients’ months after considering clinical cure [32,33]. Zhang and Tarleton (1999) [34] reported that kDNA signal was detectable in mice injected with *T*. *cruzi* kDNA (Brazil and Sylvio X10/4) after 2 days post-injection. Considering *R*. *prolixus* as a study model, Dias et al (2015) [28] demonstrated the kinetics of parasite DNA after a blood meal containing heat-killed *T*. *cruzi*. In this *in vivo* analysis, with lysed parasites, the study showed that *T cruzi* DNA remained detectable for at least 4 days after feeding with non-viable parasites, which agrees with our *in vitro* analysis. Besides that, De Oliveira et al. (2019) [35] showed that açaí samples experimentally contaminated with DNA from non-viable parasites was detectable for hours after the loss of viability and they also observed a rapid degradation of *T*. *cruzi* mRNA after açai samples were submitted to sanitization and heat treatment. Moreover, De Oliveira et al. (2021) [36] compared DNA and RNA based PCR amplifications in inoculated açai juice and fruits that were pasteurized and blanched, although there was not an internal amplification control included in neither the qPCR nor RT-qPCR, which is important to avoid false-negative results and detect variations between highly complex samples. As demonstrated in the present study, De Oliveira et al. (2021) [36] also observed differences between both molecular methodologies, in which qPCR revealed the presence of amplification signals after heat treatment whereas RT-qPCR no amplification signal in samples under same conditions [36].

Alternative molecular methods using the pathogen’s RNA to discriminate between live or dead parasites have been reported [14–16,37,38]. However, until now, few reports are comparing the application of molecular diagnostic tools that effectively differentiate between viable and non-viable parasites, especially when it comes to *T*. *cruzi* evaluation. Therefore, we developed and standardized a RT-qPCR using *T*. *cruzi* mRNA as a suitable marker to evaluate viable parasites load in *R*. *prolixus*. Regarding *T*. *cruzi* viability, this study confirmed that, while RNA is shortly degraded after parasite lysis, DNA can endure a longer time circulating and therefore a positive result does not necessarily indicate living parasites.

As reported in previously published studies [10,28], we observed that both RT-qPCR and qPCR were able to detect small quantities of parasites in the digestive tract, while microscopic examination could only count those insects with higher parasite loads (over 10^4^ parasites/mL). Besides that, due to the dark color of the examined content, there were difficulties in parasites visualization and counting on days 5 and 9 after feeding.

*T*. *cruzi* interactions along the digestive tract depend on several parameters such as insect vector species, the nature of the gut microbiota, and antimicrobial factors [7,39]. Also, *T*. *cruzi* infection can activate different types of invertebrate host’s immunity effectors molecules that are rapidly synthesized after microorganism’s invasion [40,41], affecting parasite population. Furthermore, the strain or lineage of *T*. *cruzi*, as well as the evolutive form ingested at the time of the triatomine feeding, can contribute to a vector’s susceptibility, since these differences can reflect different levels of triatomine infection [7,28,42,43]. Our data on *T*. *cruzi* (Dm28c) kinetics in *R*. *prolixus* entire digestive tract showed that, despite the significant differences in the parasite load between DNA and RNA quantification methodologies, both parasite load curves (**Fig 4**) exhibited a similar profile over the days, with a reduction of the parasite load. Henriques et al. (2012) [44] also reported this parasite load profile from the second week after *R*. *prolixus* infection with *T*. *cruzi* labeled with luciferase (Dm28c-luc). Nevertheless, our data showed a statistically significant reduction in mRNA amount on day 15 after feeding, which occurs more rapidly than the parasite load decrease observed through DNA detection.

Results concerning mRNA quantification in different portions of *R*. *prolixus* digestive tract agreed with previous studies that assessed the parasite load through bioluminescence imaging or DNA-based assays [27,28,43,44]. These results, about kinetics of *T*. *cruzi* (Dm28c) colonization in *R*. *prolixus* digestive tract, demonstrate that a significant portion of these parasites is lysed during early stages of infection. This lysis takes place in the anterior midgut portion of the digestive tract, the first interaction point between the ingested *T*. *cruzi* with bacteria of the microbiota present on the surface of the vector’s digestive tract [21,43]. After that, the remaining parasites migrate to the posterior midgut where they can attach to it and epimastigotes forms will proliferate [45–47]. Paranaiba et al. (2021) [47] also stated, through microscopic analysis, this escape from the anterior midgut portion of *Triatoma infestans* that was experimentally fed with three different *T*. *cruzi* DTUs (TcI, TcII and TcIII). Once *T*. *cruzi* reach the hindgut, these replicate forms differentiate into infective metacyclic trypomastigotes which will be eventually released in the excreta [21,48]. Taken together, our results showed a constant parasite load in the PM of *R*. *prolixus* and, while the population of viable parasites from the AM decreased significantly, the number of viable parasites in the Hindgut concomitantly increased over the days after feeding. Considering the model of *T*. *infestans* infected with TcI, TcII or TcIII, this migration of parasites from AM to PM+H was also observed occurring concomitantly [47].

According to Henriques et al. (2012) [44], trypomastigote forms were detected in the final portion of *R*. *prolixus* digestive tract from the third week after feeding. The RT-qPCR developed herein is not able to differentiate between trypomastigote and epimastigote forms with TcGAPDH target since its expression is constitutive in all *T*. *cruzi* evolutive stages. However, based on previous reports, and because *T*. *cruzi* metacyclogenesis process takes place in the final portion of the triatomines digestive tract, we can estimate that the viable parasite load observed in the hindgut corresponds mostly to metacyclic trypomastigotes. Nevertheless, the development of a molecular method capable of differentiating *T*. *cruzi* trypomastigotes and epimastigotes, through the quantification of trypomastigote forms using stage-specific genes as targets, is important to improve the diagnosis of infected triatomines. In addition, we aim to continue standardizing the technique, adapting this SYBR-green RT-qPCR to a TaqMan system, by designing specific probes for each target, along with developing a one-step methodology for less handling of RNA samples. And, although using other species of triatomines and carrying out an experiment to validate the technique with field-collected triatomines collected are a perspective, this novel RT-qPCR methodology has potential application in viability assessment and raises the possibility for further monitoring of the parasite load in infected insects or studies related to vectorial competence.

In conclusion, this first comparison analysis conducted herein showed some differences between *T*. *cruzi* DNA and RNA quantification in the kinetics of parasite colonization in *R*. *prolixus*., suggesting that RNA has the potential to be considered a marker of *T*. *cruzi* viability. Furthermore, the analysis of parasite viability, by RT-qPCR could contribute to better investigations of parasite infection dynamics, vector competence and evaluation of trypanocidal drugs. It could also contribute to food security, through the development of new molecular assays for testing food samples for pathogens, helping in oral infections studies or in outbreaks investigations.

## Supporting information

S1 Fig Representative amplification plots and melting curves for RT-qPCR assays. Assays were performed using cDNA from a pool of 3 *R*. *prolixus* digestive tubes spiked with 10^5^ *T*. *cruzi*, in a pool of 3 non-infected *R*. *prolixus* digestive tubes and from a positive control containing 10^5^ *T*. *cruzi* (Dm28c epimastigotes) solely. (A-B) Amplification plot and melt curve for TcGAPDH target. The arrows indicate the Negative Template Control (NTC) and the specificity of the primers since there was no amplification for non-infected *R*. *prolixus* sample. (C-D) Amplification plot and melt curve for the gene correspondent to the 12S region of the ribosomal RNA of triatomine. The arrows indicate the NTC and the specificity of the primers since there was no amplification a positive control sample corresponding 10^5^ *T*. *cruzi* equivalents/mL. No RT controls (-RT controls) were included in all assays. All primers sets generated a single product peak, indicating their specificities. (E-F) Amplification plots in linear scale to the TcGAPDH and 12S region of the ribosomal RNA, respectively, evidencing the ΔRn values (Y-axes) for each curve.
(TIF)
