# Supplementary figures and images for: RNA as a feasible marker of Trypanosoma cruzi viability during the parasite interaction with the triatomine vector Rhodnius prolixus (Hemiptera, Triatominae)
Source: PLoS Negl Trop Dis. 2022 Jul 7;16(7):e0010535. doi: 10.1371/journal.pntd.0010535 (PMC9307183; doi:10.1371/journal.pntd.0010535)

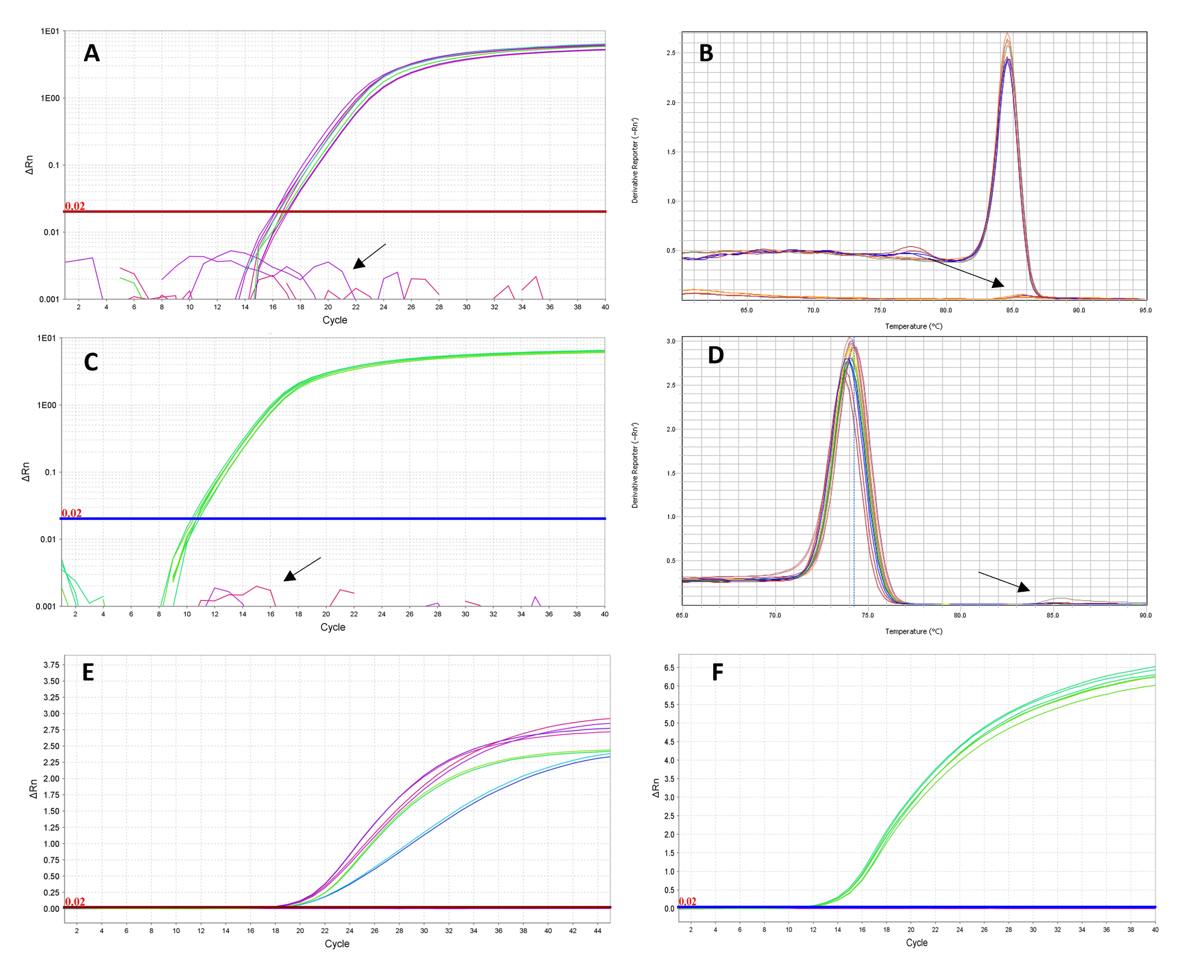

Supplement: S1 Fig — Assays were performed using cDNA from a pool of 3 R. prolixus digestive tubes spiked with 105 T. cruzi, in a pool of 3 non-infected R. prolixus digestive tubes and from a positive control containing 105 T. cruzi (Dm28c epimastigotes) solely. (A-B) Amplification plot and melt curve for TcGAPDH target. The arrows indicate the Negative Template Control (NTC) and the specificity of the primers since there was no amplification for non-infected R. prolixus sample. (C-D) Amplification plot and melt curve for the gene correspondent to the 12S region of the ribosomal RNA of triatomine. The arrows indicate the NTC and the specificity of the primers since there was no amplification a positive control sample corresponding 105 T. cruzi equivalents/mL. No RT controls (-RT controls) were included in all assays. All primers sets generated a single product peak, indicating their specificities. (E-F) Amplification plots in linear scale to the TcGAPDH and 12S region of the ribosomal RNA, respectively, evidencing the ΔRn values (Y-axes) for each curve. (TIF) [file pntd.0010535.s001.tif]
